# Higher Responsiveness for Women, High Transaminase Levels, and Fat Percentage to Pemafibrate Treatment for NAFLD

**DOI:** 10.3390/biomedicines10112806

**Published:** 2022-11-04

**Authors:** Takanobu Iwadare, Takefumi Kimura, Hideo Kunimoto, Naoki Tanaka, Shun-ichi Wakabayashi, Tomoo Yamazaki, Taiki Okumura, Hiroyuki Kobayashi, Yuki Yamashita, Ayumi Sugiura, Satoru Joshita, Takeji Umemura

**Affiliations:** 1 Department of Medicine, Division of Gastroenterology and Hepatology, Shinshu University School of Medicine, Matsumoto 390-8621, Japan; 2 Consultation Center for Liver Diseases, Shinshu University Hospital, Matsumoto 390-8621, Japan; 3 Department of Gastroenterology, Nagano Municipal Hospital, Nagano 381-8551, Japan; 4 Department of Global Medical Research Promotion, Shinshu University Graduate School of Medicine, Matsumoto 390-8621, Japan; 5 International Relations Office, Shinshu University School of Medicine, Matsumoto 390-8621, Japan; 6 Department of Medicine, University of California San Diego, La Jolla, CA 92037, USA

**Keywords:** non-alcoholic fatty liver disease, non-alcoholic steatohepatitis, hypertriglyceridemia, pemafibrate, selective PPARα modulator, treatment prediction, body composition analysis

## Abstract

Aim: Pemafibrate (PEM) is a novel selective peroxisome proliferator-activated receptor alpha modulator that is effective for hypertriglyceridemia accompanying non-alcoholic fatty liver disease (HTG-NAFLD). This study aimed to identify the predictors of PEM efficacy for HTG-NAFLD in clinical practice. Methods: We retrospectively enrolled 88 HTG-NAFLD patients treated with PEM for 6 months for the analysis of routine blood and body composition testing. A PEM response was defined as a decrease in serum alanine aminotransferase (ALT) of >30% compared with pre-treatment level. The clinical features related to PEM responsiveness were statistically tested between responders and non-responders. Results: All 88 patients completed the 6 month drug regimen without any adverse effects. PEM treatment significantly decreased liver enzymes, triglycerides, and total cholesterol levels, without any detectable impact on body weight or body composition. Comparisons of baseline clinical features revealed female and greater aspartate aminotransferase (AST), ALT, and fat mass % levels to be significantly associated with a PEM response. The optimal cut-off values to predict responders as determined by receiver operating characteristic analysis were AST 45 U/L, ALT 60 U/L, and fat mass 37%. Conclusions: Female HTG-NAFLD patients with higher transaminase and fat mass % levels may be preferentially indicated for PEM treatment. Additional large-scale prospective studies are warranted to verify our results.

## 1. Introduction

Non-alcoholic fatty liver disease (NAFLD) is considered a hepatic manifestation of metabolic syndrome and is related to such metabolic diseases as obesity, hypertension (HT), type 2 diabetes mellitus (DM), and dyslipidemia [1]. NAFLD is also the most common chronic liver disease, whose incidence is increasing worldwide [2]. NAFLD is classified into non-alcoholic fatty liver and non-alcoholic steatohepatitis (NASH) [3]. NASH increases the risk of liver cirrhosis (LC), liver failure, and hepatocellular carcinoma (HCC) [4]. Despite intensive treatment development efforts, no US Food and Drug Administration approved drug therapy is currently available for human NAFLD/NASH [5].

Pemafibrate (PEM) is a novel selective peroxisome proliferator-activated receptor-alpha (PPARα) modulator approved for use in Japan in 2018 [6]. The clinical dose of PEM is thought to reduce triglyceride (TG) levels by up-regulating hepatic PPARα activity [7]. As PPARα is considered to have a hepatoprotective function [8], therapeutic strategies to restore PPARα expression/activity may be beneficial [9,10]. Raza-Iqbal et al. showed that PEM induced a series of PPARα target genes involved in hepatic TG hydrolysis, fatty acid uptake, fatty acid β-oxidation, and ketogenesis, all of which underpin the drug’s plasma TG-lowering effects [11].

Although the usefulness of PEM in NAFLD patients has been described in recent years, the predictors of treatment efficacy have not yet been investigated. This study evaluated the clinical and body composition features predicting a PEM response for hypertriglyceridemia accompanying NAFLD (HTG-NAFLD).

## 2. Materials and Methods

### 2.1. Patients and Clinical Examinations

This retrospective, case-control study was approved by the Committees for Medical Ethics of Shinshu University School of Medicine (ID number: 4196) and Nagano Municipal Hospital (ID number: 0038), and was performed following the Helsinki declaration of 1975, 1983 revision. We carefully reviewed the medical records of 30 Japanese NAFLD patients who had visited Shinshu University Hospital (Matsumoto, Japan) between January 2019 and June 2022, and 58 Japanese NAFLD patients who had been treated at Nagano Municipal Hospital (Nagano, Japan) between August 2019 and May 2022. HTG-NAFLD was diagnosed based on the following criteria: (1) the presence of hepatorenal contrast and increased hepatic echogenicity on abdominal ultrasonography; (2) an average habitual consumption of <20 g/day of ethanol; (3) the absence of other causes of liver dysfunction, such as viral hepatitis, drug-induced liver injury, autoimmune liver disease, Wilson’s disease, hereditary hemochromatosis, and citrin deficiency [12], and (4) fasting serum levels of TG > 150 mg/dL [13]. All patients had well-preserved liver function (i.e., not Child-Pugh class B or C) and no signs of HCC, gallstones, or advanced renal impairment (i.e., serum creatine (Cre) concentration ≥2.5 mg/dL) before PEM medication. Patients were defined as hypertensive if their systolic/diastolic pressure was >140/90 mmHg or if they were taking anti-hypertensive drugs [14]. Patients were considered to be diabetic if they had a fasting glucose level of ≥126 mg/dL or if they were taking insulin or oral hypoglycemic agents [15]. The diagnosis of LC was based on imaging findings and the formula for predicting LC proposed by Ikeda et al. [16]. All laboratory data and body composition measurements were obtained in a fasting state. Fibrosis-4 index (FIB-4) and aspartate aminotransferase (AST) to platelet ratio index (APRI) were calculated according to the following formulas: FIB-4 = (age [years] × AST [U/L])/(platelet count (PLT) [×10^9^/L] × alanine aminotransferase (ALT) [U/L]^1/2^) [17], and APRI = AST [U/L]/upper limit of the normal range [U/L]/PLT [10^9^/L] × 100 [18,19]. The interval between patient visits and blood sampling was 4 weeks, at which time the patient was also interviewed about side effects.

### 2.2. Body Composition Analysis

For the 58 patients seen at Nagano Municipal Hospital, body composition analysis to measure fat mass, soft lean mass, and skeletal muscle (SKM) mass, was performed using an InBodyS10 multifrequency impedance body composition analyzer (InBody, Tokyo, Japan). Skeletal muscle mass index (SMI) was calculated as appendicular SKM mass [kg]/height [m]^2^. Referring to the Japanese Society of Hepatology sarcopenia diagnostic criteria, we defined sarcopenia as SMI < 7.0 kg/m^2^ in men and <5.7 kg/m^2^ in women [20].

### 2.3. Statistical Analysis

Clinical data are expressed as the number (percentage) or median (interquartile range). Statistical analyses were performed using the StatFlex Ver. 7.0 (Artech. Co., Ltd., Osaka, Japan) and Prism 8 (GraphPad Software Inc.; San Diego, CA, USA). Wilcoxon matched-pairs signed-rank testing was used for evaluating parameters before and after PEM treatment. The Mann–Whitney test and chi-square test were employed to compare responders and non-responders. Correlation analysis was conducted using Spearman’s test. Diagnostic accuracy was evaluated using the area under the receiver operating characteristic (ROC) curve (AUC). The Youden index identified cut-off values, with the nearest clinically applicable value to the cut-off being considered the optimal threshold for clinical convenience. All statistical tests were evaluated at the 0.05 level of significance.

## 3. Results

### 3.1. Clinical Characteristics of HTG-NAFLD Patients Treated with PEM

We ultimately enrolled 88 patients receiving PEM (0.1 mg twice daily) for HTG-NAFLD and examined the effects of PEM treatment for 6 months. The pretreatment clinical characteristics of the cohort are summarized in Table 1. Median age was 57 years, and 35 patients (39.8%) were men. Body composit.ion analysis revealed median body weight, body mass index (BMI), fat mass percentage (%), and SMI values of 71.9 kg, 27.2 kg/m^2^, 39%, and 7.4 kg/m^2^, respectively. Forty-one (47%) and 31 (35%) patients had DM and HT, respectively.

### 3.2. Six-Month Treatment with PEM Significantly Improved Liver Function and Lipid Profiles

**Figure 1:**
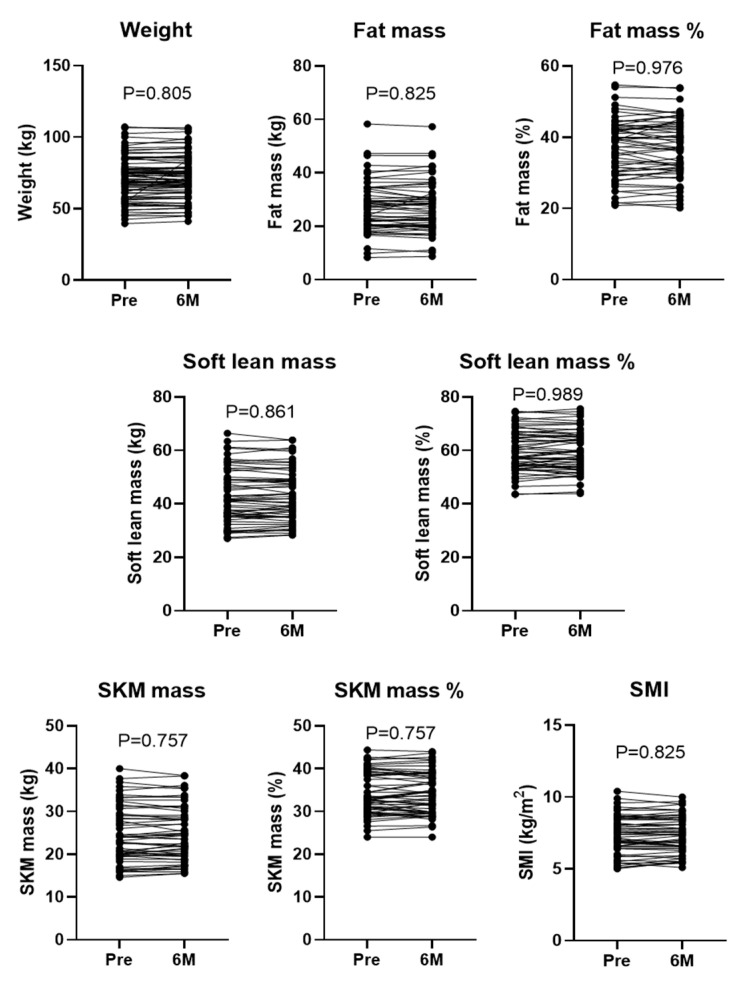
Physical data were evaluated before (Pre) and at 6 months (6 M) of PEM treatment. Data are from 58 patients for body composition analysis, and 88 patients for weight (Wilcoxon matched-pairs signed-rank test). SKM: skeletal muscle, SMI: skeletal muscle mass index.

**Figure 2:**
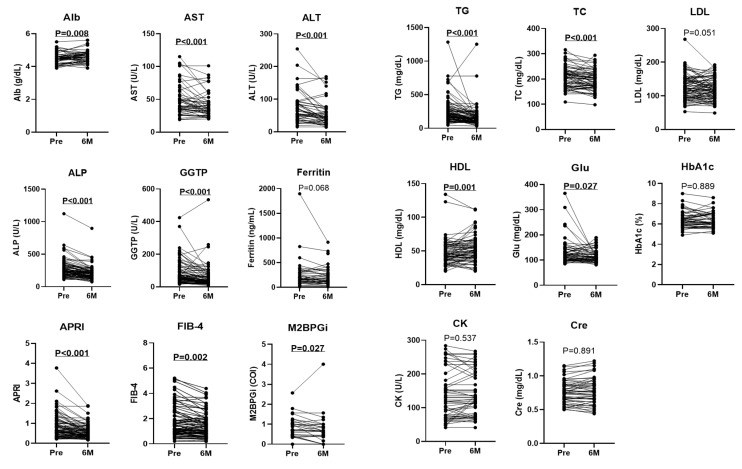
Laboratory data were evaluated before (Pre) and at 6 months (6 M) of PEM treatment. Data are from 47 patients for ferritin, 31 patients for M2BPGi, and 88 patients for the other parameters (Wilcoxon matched-pairs signed-rank test). Alb: albumin, ALP: alkaline phosphatase, ALT: alanine aminotransferase, APRI: aspartate aminotransferase to platelet ratio index, AST: aspartate aminotransferase, CK: creatine kinase, Cre: creatinine, FIB-4: fibrosis-4 index, GGTP: gamma-glutamyltransferase, Glu: fasting glucose, HbA1c: hemoglobin A1c, HDL: high-density lipoprotein cholesterol, LDL: low-density lipoprotein cholesterol, M2BPGi: Mac-2 binding protein glycan isomer, TC: total cholesterol, TG: triglyceride.

We analyzed the changes in clinical parameters before and at 6 months of PEM treatment in patients with HTG-NAFLD. No significant treatment-induced changes were seen for body composition, including body weight, fat mass %, and SMI (Figure 1). The median values of AST, ALT, alkaline phosphatase (ALP), and gamma-glutamyltransferase (GGTP) were all significantly improved at 6 months compared with baseline (AST: 43 to 34 U/L, ALT: 56 to 38 U/L, ALP: 241 to 189 U/L, and GGTP: 59 to 33 U/L, all *p* < 0.001) (Figure 2). Twenty-five patients (28.4%) had both AST and ALT below 40 U/L before PEM treatment, which increased to 41 (46.6%) after treatment. A significant increase in serum albumin (Alb) was also detected (4.4 to 4.6 g/dL, *p* = 0.008). Regarding liver fibrosis markers, APRI, FIB-4, and Mac-2 binding protein glycan isomer (M2BPGi) values at 6 months were significantly lower than at baseline (APRI: 0.7 to 0.49, *p* < 0.001, FIB-4: 1.21 to 1.18, *p* = 0.002 and M2BPGi: 0.81 to 0.7 COI, *p* = 0.027).

Lipid profiles including TG, total cholesterol (TC), and high-density lipoprotein cholesterol (HDL) were improved at 6 months compared with baseline, as previously reported (TG: 197 to 128 mg/dL, *p* < 0.001, TC: 209 to 194 mg/dL, *p* < 0.001, and HDL: 43 to 51 mg/dL, *p* = 0.001), whereas low-density lipoprotein cholesterol tended to be comparable (124 to 115 mg/dL, *p* = 0.051) (Figure 2). Fasting glucose level was decreased at 6 months of PEM treatment (117 to 111 mg/dL, *p* = 0.027), while hemoglobin A1c remained unchanged (6.3% to 6.0%, *p* = 0.889). No significant differences in creatine kinase or Cre values before and after treatment were noted. All patients were able to continue PEM treatment without noticeable adverse effects.

### 3.3. Comparison between Responders and Non-Responders to PEM Treatment in HTG-NAFLD Patients

**Figure 3:**
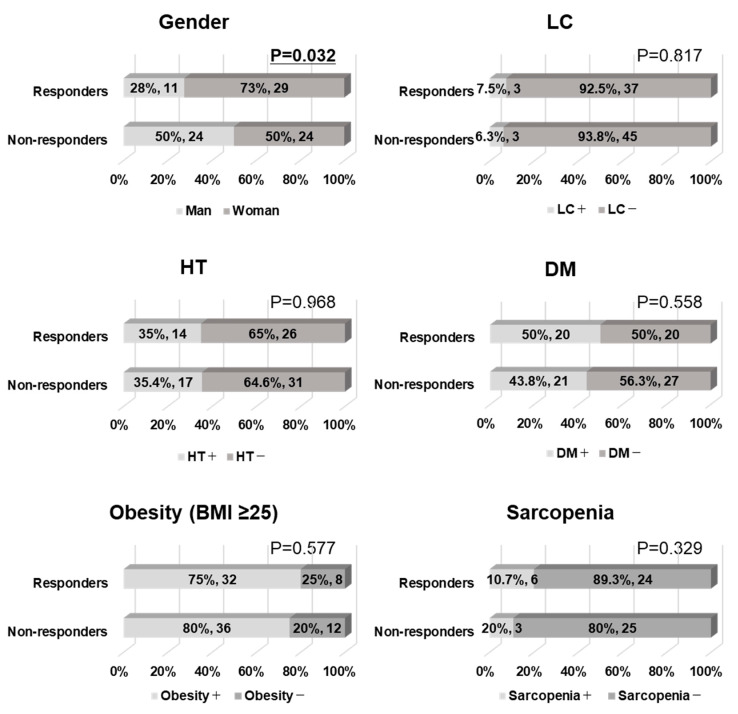
Patient background comparison of responders and non-responders to 6 months of PEM treatment. Patients were defined as responders if ALT decreased by >30% at 6 months of PEM treatment (Chi-square test). Sarcopenia is defined in the Methods section. BMI: body mass index, DM: diabetes mellitus, HT: hypertension, LC: liver cirrhosis.

A response to pharmacological intervention was defined as a decrease in ALT of >30% compared with baseline after 6 months of PEM treatment according to several NAFLD clinical trials [21,22]. We compared the baseline clinical features of PEM responders (n = 40) and non-responders (n = 48) in HTG-NAFLD patients, including those who underwent body composition analysis. Responders had a significantly higher frequency of women than non-responders (73% vs. 50%, *p* = 0.032) (Figure 3). The presence of LC, HT, DM, obesity (i.e., BMI ≥ 25), or sarcopenia did not significantly affect PEM treatment response.

**Figure 4:**
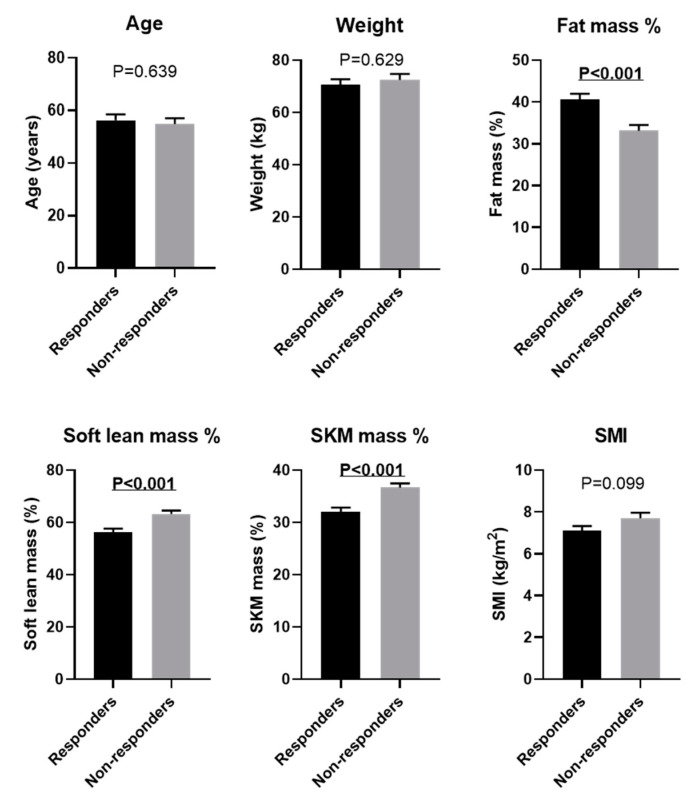
Comparison of baseline physical data of responders and non-responders to 6 months of PEM treatment (Mann–Whitney U test). SKM: skeletal muscle, SMI: skeletal muscle mass index.

**Figure 5:**
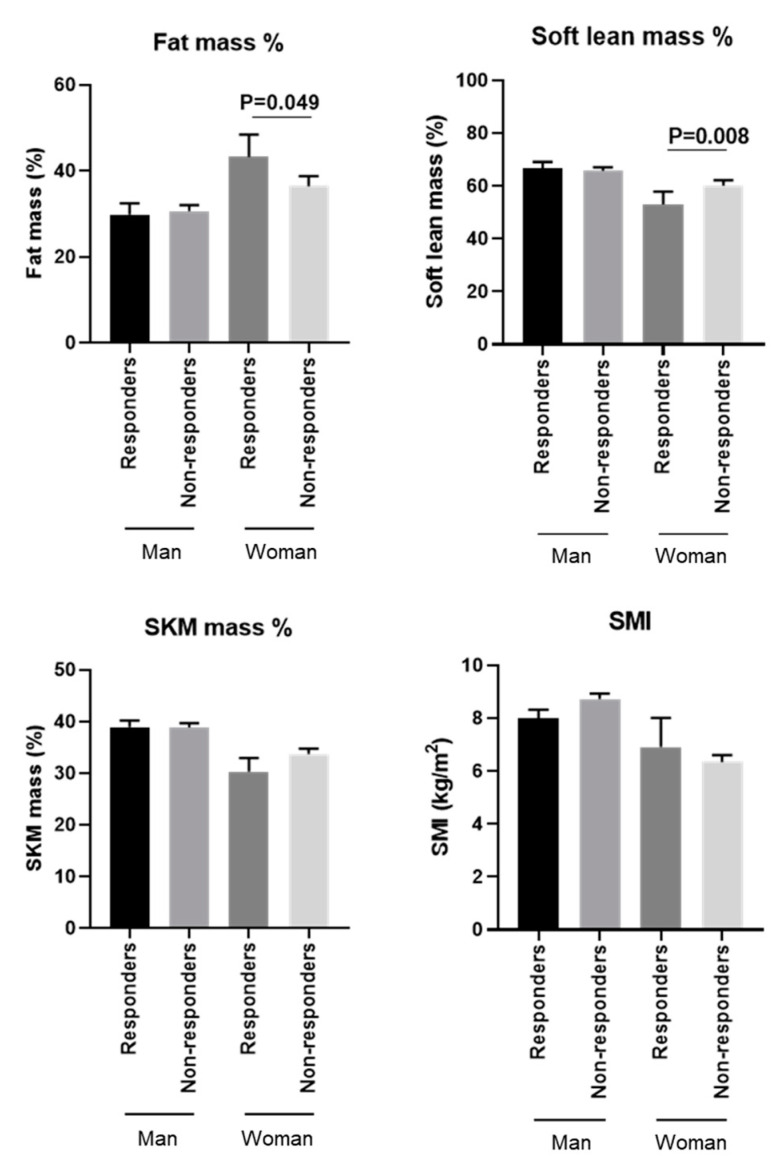
Comparison of gender-based baseline body composition analysis of responders and non-responders to PEM treatment (Mann–Whitney U test). SKM: skeletal muscle, SMI: skeletal muscle mass index.

**Figure 6:**
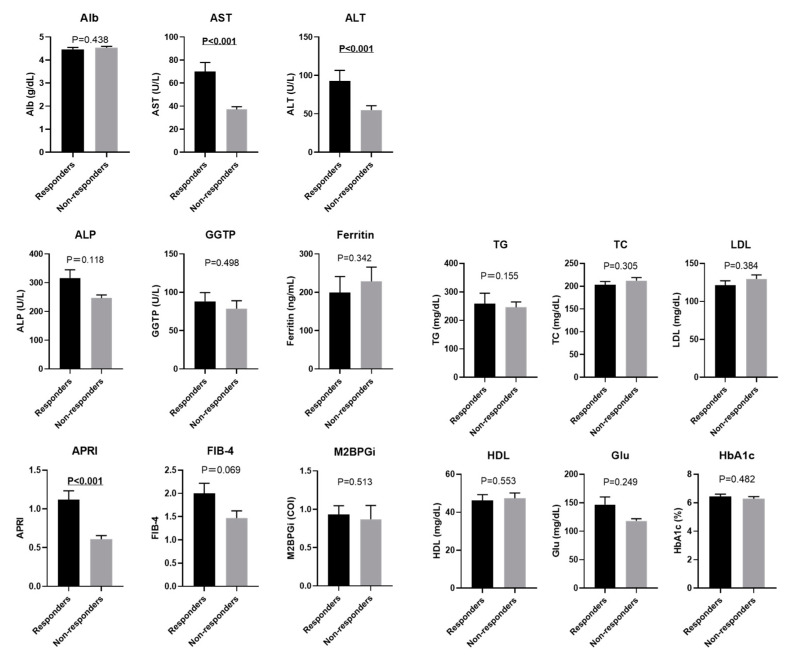
Comparison of baseline laboratory data of responders and non-responders to 6 months of PEM treatment (Mann–Whitney U test). Alb: albumin, ALP: alkaline phosphatase, ALT: alanine aminotransferase, APRI: aspartate aminotransferase to platelet ratio index, AST: aspartate aminotransferase, FIB-4 index: fibrosis based on four factors index, GGTP: gamma-glutamyltransferase, Glu: fasting glucose, HbA1c: hemoglobin A1c, HDL: high-density lipoprotein cholesterol, LDL: low-density lipoprotein cholesterol, M2BPGi: Mac-2 binding protein glycan isomer, TG: triglyceride, TC: total cholesterol.

The clinical parameters of responders and non-responders before PEM treatment were compared next. Body composition analysis revealed that responders had significantly higher fat mass %, lower soft lean mass %, and lower SKM mass % before starting PEM treatment as compared with non-responders (all *p* < 0.001) (Figure 4). There was a trend towards better treatment efficacy in patients with lower SMI, although this did not reach significance (*p* = 0.099). Focusing on each gender, we observed a significant difference in fat mass % and soft lean mass % between responders and non-responders in women (fat mass %: *p* = 0.049, and soft lean mass %: *p* = 0.008), but not in men (Figure 5). Responders also had significantly higher levels of AST, ALT, and APRI before PEM treatment compared with responders (all *p* < 0.001). Lipid profile did not remarkably affect PEM treatment response (Figure 6).

### 3.4. ROC Curve Analysis

The respective AUC values for AST, ALT, and fat mass % were 0.80, 0.72, and 0.77. The most appropriate ROC cut-off values for those parameters for discriminating between responders and non-responders were identified as AST: 45 U/L (sensitivity: 70%, and specificity: 77%), ALT: 60 U/L (sensitivity: 70%, and specificity: 71%), and fat mass: 37% (sensitivity: 77%, and specificity: 71%).

## 4. Discussion

This retrospective study evaluated the efficacy and responsiveness of PEM in patients with dyslipidemia NAFLD. Six months of PEM treatment produced the desired metabolic changes of decreased AST, ALT, GGTP, ALP, APRI, FIB-4, M2BPGi, TG, TC, and fasting glucose, as well as increasing Alb and HDL, without any clinically meaningful changes in weight or body composition.

PEM has been reported to improve liver dysfunction in patients with HTG-NAFLD in seven recent papers, as summarized in Table 2. Seko et al. witnessed that PEM significantly improved ALT in addition to TG and HDL in 20 NAFLD patients with dyslipidemia [23]. Shinozaki et al. described that markers of hepatic inflammation, function, and fibrosis, improved after 3 and 12 months of PEM treatment [24,25]. Hatanaka et al. showed dramatic transaminase level improvement in 31 biopsy-proven NASH patients with significant disease activity and advanced fibrosis [26]. They also observed ameliorated FibroScan-AST score in 10 patients with NAFLD during PEM treatment, which correlated with ALT changes and demonstrated the hepatic anti-inflammatory effect of PEM [27]. Ikeda et al. reported that PEM significantly decreased TG and ALT in 16 NAFLD patients, with fatty liver improvement in some cases [28]. Nakajima et al. evaluated the efficacy and safety of PEM in 58 patients with NAFLD in a placebo-controlled study and found that while treatment for 18 months did not reduce liver fat content, it significantly decreased magnetic resonance elastography-measured liver stiffness [29]. In the present investigation, 6 months of PEM treatment improved lipid markers as well as liver function, including AST, ALT, ALP, and GGTP, in agreement with earlier reports. A novel finding in this study was that female gender, higher pre-treatment AST, ALT, APRI, and body fat %, and lower soft lean mass % and SKM % were factors significantly associated with a response to PEM treatment. To our knowledge, this is the first report incorporating body composition analysis to predict the response to PEM therapy in patients with HTG-NAFLD.

High body fat mass %, low soft lean mass %, and low SKM mass % have been reported as risk factors for physical activity impairment and diminished clinical outcome [30]. Araki et al. found that PEM induced adipose triglyceride lipase and hormone-sensitive lipase expression in epididymal white adipose tissue, leading to the activation of lipolysis in adipocytes [31]. Enhanced mobilization of fatty acids from white adipose tissue to the liver for ensuing β-oxidation might therefore be associated with NAFLD improvement, which could at least partially explain the high efficacy of PEM in NAFLD patients with high body fat mass %.

Lastly, a recent mouse study by Smati et al. uncovered a marked sex difference in hepatic gene alterations in diet-induced NAFLD, in which PPARα played an important role [32]. Similarly, the response to PEM treatment our cohort was higher in female than in male patients, thus supporting that a determinant of this response was PPARα in hepatocytes. Molecular signatures in the human liver also indicated a sex-differentiated gene expression profile and a sex-specific co-expression network of PPARα [32]. The high response of women to PEM in our study may be attributed to the sex differences in this PPARα co-expression network in the liver. It might even be argued that the gender difference in PEM responsiveness was the main contributing factor, while the difference in body composition was a singular cofounding one. However, as shown in Figure 5, there were significant differences in treatment responsiveness according to body composition even among female patients, and so variability in body composition might be meaningful when considering PEM treatment responsiveness.

This study had several limitations in that it was retrospective in nature and of a limited cohort size. Further large-scale, prospective studies are required to confirm our results. In addition, the response to PEM treatment was evaluated in this study by a 30% reduction in ALT. However, this method of evaluation is not absolute and should be validated with an evaluation that includes a liver biopsy. Moreover, it is also necessary information whether the results from the multi-frequency impedance body composition analyzer used in this study are the same as those obtained using MRI or dual-energy x-ray absorptiometry.

In summary, this investigation demonstrated significant improvements in liver function with PEM treatment in HTG-NAFLD patients. Through the inclusion of body composition analysis, we uncovered that female gender, higher pre-treatment AST, ALT, APRI, and body fat mass %, and lower soft lean mass % and SKM mass % were significantly associated with a PEM response, all of which might represent treatment indicators in HTG-NAFLD patients.

## Figures and Tables

**Table 1:** Clinical characteristics of HTG-NAFLD patients treated with PEM.

	All Patients (n = 88)			All Patients (n = 88)	
	Median (IQR)/n (%)			Median (IQR)	
Age (years)	57	(46–66)	**Laboratory data**		
Man	35	(39.8)	Alb (g/dL)	4.4	(4.2–4.6)
Liver cirrhosis	6	(6.8)	AST (U/L)	43	(30–61)
			ALT (U/L)	56	(37–85)
**Body composition**			ALP (U/L)	241	(198–325)
Body weight (kg)	71.9	(61–79.3)	GGTP (U/L)	59	(40–95)
BMI (kg/m^2^)	27.2	(25.2–30.3)	Ferritin (ng/mL) ^$^	163	(87–278)
Fat mass (%) ^#^	39	(30.4–42.6)	APRI	0.7	(0.4–1.1)
Soft lean mass (%) ^#^	57.7	(54–66)	FIB-4	1.21	(0.89–2.45)
SKM mass (%) ^#^	33.1	(30.5–38.9)	M2BPGi (COI) *	0.81	(0.47–1.24)
SMI (kg/m^2^) ^#^	7.4	(6.6–8.5)	TG (mg/dL)	197	(153–288)
			TC (mg/dL)	209	(173–233)
**Complications**			LDL (mg/dL)	124	(99–150)
Type 2 DM	41	(47%)	HDL (mg/dL)	43	(36–656)
Hypertension	31	(35%)	Fasting glucose (mg/dL)	117	(104–137)
Obesity (BMI ≥ 25)	68	(77%)	HbA1c (%)	6.3	(5.9–6.6)
Sarcopenia ^#^	29	(50%)	Cre (mg/dL)	0.75	(0.65–0.89)
			CK (U/L)	102	(76–174)

^#^ n = 58, ^$^ n = 47, * n = 31. Alb: albumin, ALP: alkaline phosphatase, ALT: alanine aminotransferase, APRI: AST to platelet ratio index, AST: aspartate aminotransferase, BMI: body mass index, CK: creatine kinase, Cre: creatinine, DM: diabetes mellitus, FIB-4: fibrosis-4 index, GGTP: gamma-glutamyltransferase, HbA1c: hemoglobin A1c, HDL: high-density lipoprotein cholesterol, HTG-NAFLD: hypertriglyceridemia accompanying non-alcoholic fatty liver disease, IQR: interquartile range, LDL: low-density lipoprotein cholesterol, M2BPGi: Mac-2 binding protein glycan isomer, PEM: pemafibrate, SKM: skeletal muscle, SMI: skeletal muscle mass index, TC: total cholesterol, TG: triglyceride.

**Table 2:** Reports on pemafibrate treatment for patients with NAFLD.

Author	Year	No. of Cases	Drug Dose (Twice Daily)	Study Design	Pre ALT (U/L)	3M ALT (U/L)	6M ALT (U/L)	12M ALT (U/L)
**Our study**	**2022**	**88**	**0.1 mg**	**Retrospective**	56 *	**NS**	**38**	**NS**
Nakajima et al. [29]	2021	58	0.2 mg	Double-blind RCT	83 ^†^	NS	50	48
Shinozaki et al. [25]	2021	22	0.1 mg	Retrospective	65 ^†^	NS	NS	30
Ikeda et al. [28]	2021	16	0.1–0.2 mg	Retrospective	65 *	NS	NS	28
Hatanaka et al. [26]	2021	31	0.1 mg	Retrospective	49 *	33	25	32
Hatanaka et al. [27]	2021	10	0.1 mg	Retrospective	52 *	32	23	NS
Shinozaki et al. [24]	2020	38	0.1 mg	Retrospective	64 ^†^	42	NS	NS
Seko et al. [23]	2020	20	0.1 mg	Prospective	75 ^†^	44	NS	NS

Apart from the Nakajima et al. study, all NAFLD cases were with hypertriglyceridemia or/and dyslipidemia. Values are indicated by * for the median and † for the mean. ALT: alanine aminotransferase, M: months, NAFLD: non-alcoholic fatty liver disease, NS: not significant, RCT: randomized controlled trial.

## Data Availability

Study data will be provided by the corresponding author upon reasonable request.

## Abbreviations

Alb: albumin, ALP: alkaline phosphatase, ALT: alanine aminotransferase, APRI: aspartate aminotransferase to platelet ratio index, AST: aspartate aminotransferase, AUC: area under the receiver operating characteristic curve, BMI: body mass index, Cre: creatinine, DM: diabetes mellitus, FIB-4: fibrosis-4 index, GGTP: gamma-glutamyltransferase, HCC: hepatocellular carcinoma, HDL: high-density lipoprotein cholesterol, HT: hypertension, HTG: hypertriglyceridemia, HTG-NAFLD: hypertriglyceridemia accompanying non-alcoholic fatty liver disease, LC: liver cirrhosis, M2BPGi: Mac-2 binding protein glycan isomer, NAFLD: non-alcoholic fatty liver disease, NASH: non-alcoholic steatohepatitis, PEM: pemafibrate, PLT: platelet count, PPARα: peroxisome proliferator-activated receptor-alpha, ROC: receiver operating characteristic, SKM: skeletal muscle, SMI: skeletal muscle mass index, TC: total cholesterol, TG: triglyceride.

## References

[1] Angulo P. (2002). Nonalcoholic fatty liver disease. N. Engl. J. Med..

[2] Younossi Z., Anstee Q.M., Marietti M., Hardy T., Henry L., Eslam M., George J., Bugianesi E. (2018). Global burden of NAFLD and NASH: Trends, predictions, risk factors and prevention. Nat. Rev. Gastroenterol. Hepatol..

[3] Friedman S.L., Neuschwander-Tetri B.A., Rinella M., Sanyal A.J. (2018). Mechanisms of NAFLD development and therapeutic strategies. Nat. Med..

[4] Chalasani N., Younossi Z., Lavine J.E., Charlton M., Cusi K., Rinella M., Harrison S.A., Brunt E.M., Sanyal A.J. (2018). The diagnosis and management of nonalcoholic fatty liver disease: Practice guidance from the American Association for the Study of Liver Diseases. Hepatology.

[5] Kimura T., Singh S., Tanaka N., Umemura T. (2021). Role of G Protein-Coupled Receptors in Hepatic Stellate Cells and Approaches to Anti-Fibrotic Treatment of Non-Alcoholic Fatty Liver Disease. Front. Endocrinol..

[6] Aomura D., Harada M., Yamada Y., Nakajima T., Hashimoto K., Tanaka N., Kamijo Y. (2021). Pemafibrate Protects against Fatty Acid-Induced Nephropathy by Maintaining Renal Fatty Acid Metabolism. Metabolites.

[7] Zhang Z., Diao P., Zhang X., Nakajima T., Kimura T., Tanaka N. (2022). Clinically Relevant Dose of Pemafibrate, a Novel Selective Peroxisome Proliferator-Activated Receptor α Modulator (SPPARMα), Lowers Serum Triglyceride Levels by Targeting Hepatic PPARα in Mice. Biomedicines.

[8] Wang Y., Nakajima T., Gonzalez F.J., Tanaka N. (2020). PPARs as metabolic regulators in the liver: Lessons from liver-specific PPAR-null mice. Int. J. Mol. Sci..

[9] Tanaka N., Aoyama T., Kimura S., Gonzalez F.J. (2017). Targeting nuclear receptors for the treatment of fatty liver disease. Pharmacol. Ther..

[10] Honda Y., Kessoku T., Ogawa Y., Tomeno W., Imajo K., Fujita K., Yoneda M., Takizawa T., Saito S., Nagashima Y. (2017). Pemafibrate, a novel selective peroxisome proliferator-activated receptor alpha modulator, improves the pathogenesis in a rodent model of nonalcoholic steatohepatitis. Sci. Rep..

[11] Raza-Iqbal S., Tanaka T., Anai M., Inagaki T., Matsumura Y., Ikeda K., Taguchi A., Gonzalez F.J., Sakai J., Kodama T. (2015). Transcriptome analysis of K-877 (a novel selective PPARα modulator (SPPARMα))-regulated genes in primary human hepatocytes and the mouse liver. J. Atheroscler. Thromb..

[12] Komatsu M., Kimura T., Yazaki M., Tanaka N., Yang Y., Nakajima T., Horiuchi A., Fang Z.-Z., Joshita S., Matsumoto A. (2015). Steatogenesis in adult-onset type II citrullinemia is associated with down-regulation of PPARα. Biochim. Biophys. Acta (BBA)-Mol. Basis Dis..

[13] Simha V. (2020). Management of hypertriglyceridemia. BMJ.

[14] Kimura T., Shinji A., Horiuchi A., Tanaka N., Nagaya T., Shigeno T., Nakamura N., Komatsu M., Umemura T., Arakura N. (2012). Clinical characteristics of young-onset ischemic colitis. Dig. Dis. Sci..

[15] Kimura T., Shinji A., Tanaka N., Koinuma M., Yamaura M., Nagaya T., Joshita S., Komatsu M., Umemura T., Horiuchi A. (2017). Association between lower air pressure and the onset of ischemic colitis: A case-control study. Eur. J. Gastroenterol. Hepatol..

[16] Ikeda K., Saitoh S., Kobayashi M., Suzuki Y., Tsubota A., Suzuki F., Arase Y., Murashima N., Chayama K., Kumada H. (2000). Distinction between chronic hepatitis and liver cirrhosis in patients with hepatitis C virus infection. Practical discriminant function using common laboratory data. Hepatol. Res..

[17] Vallet-Pichard A., Mallet V., Nalpas B., Verkarre V., Nalpas A., Dhalluin-Venier V., Fontaine H., Pol S. (2007). FIB-4: An inexpensive and accurate marker of fibrosis in HCV infection. comparison with liver biopsy and fibrotest. Hepatology.

[18] Wai C.-T., Greenson J.K., Fontana R.J., Kalbfleisch J.D., Marrero J.A., Conjeevaram H.S., Lok A.S.-F. (2003). A simple noninvasive index can predict both significant fibrosis and cirrhosis in patients with chronic hepatitis C. Hepatology.

[19] Fujimori N., Kimura T., Tanaka N., Yamazaki T., Okumura T., Kobayashi H., Wakabayashi S.I., Yamashita Y., Sugiura A., Pham J. (2022). 2-Step PLT16-AST44 method: Simplified liver fibrosis detection system in patients with non-alcoholic fatty liver disease. Hepatol. Res..

[20] Nishikawa H., Shiraki M., Hiramatsu A., Moriya K., Hino K., Nishiguchi S. (2016). Japan Society of Hepatology guidelines for sarcopenia in liver disease: Recommendation from the working group for creation of sarcopenia assessment criteria. Hepatol. Res..

[21] Seko Y., Sumida Y., Tanaka S., Mori K., Taketani H., Ishiba H., Hara T., Okajima A., Yamaguchi K., Moriguchi M. (2015). Serum alanine aminotransferase predicts the histological course of non-alcoholic steatohepatitis in Japanese patients. Hepatol. Res..

[22] Nogami A., Yoneda M., Kobayashi T., Kessoku T., Honda Y., Ogawa Y., Suzuki K., Tomeno W., Imajo K., Kirikoshi H. (2019). Assessment of 10-year changes in liver stiffness using vibration-controlled transient elastography in non-alcoholic fatty liver disease. Hepatol. Res..

[23] Seko Y., Yamaguchi K., Umemura A., Yano K., Takahashi A., Okishio S., Kataoka S., Okuda K., Moriguchi M., Okanoue T. (2020). Effect of pemafibrate on fatty acid levels and liver enzymes in non-alcoholic fatty liver disease patients with dyslipidemia: A single-arm, pilot study. Hepatol. Res..

[24] Shinozaki S., Tahara T., Lefor A.K., Ogura M. (2020). Pemafibrate decreases markers of hepatic inflammation in patients with non-alcoholic fatty liver disease. Clin. Exp. Hepatol..

[25] Shinozaki S., Tahara T., Lefor A.K., Ogura M. (2021). Pemafibrate improves hepatic inflammation, function and fibrosis in patients with non-alcoholic fatty liver disease: A one-year observational study. Clin. Exp. Hepatol..

[26] Hatanaka T., Kakizaki S., Saito N., Nakano Y., Nakano S., Hazama Y., Yoshida S., Hachisu Y., Tanaka Y., Kashiwabara K. (2021). Impact of Pemafibrate in Patients with Hypertriglyceridemia and Metabolic Dysfunction-associated Fatty Liver Disease Pathologically Diagnosed with Non-alcoholic Steatohepatitis: A Retrospective, Single-arm Study. Intern. Med..

[27] Hatanaka T., Kosone T., Saito N., Takakusagi S., Tojima H., Naganuma A., Takagi H., Uraoka T., Kakizaki S. (2021). Effect of 48-week pemafibrate on non-alcoholic fatty liver disease with hypertriglyceridemia, as evaluated by the FibroScan-aspartate aminotransferase score. JGH Open.

[28] Ikeda S., Sugihara T., Kihara T., Matsuki Y., Nagahara T., Takata T., Kitao S., Okura T., Yamamoto K., Isomoto H. (2021). Pemafibrate Ameliorates Liver Dysfunction and Fatty Liver in Patients with Non-Alcoholic Fatty Liver Disease with Hypertriglyceridemia: A Retrospective Study with the Outcome after a Mid-Term Follow-Up. Diagnostics.

[29] Nakajima A., Eguchi Y., Yoneda M., Imajo K., Tamaki N., Suganami H., Nojima T., Tanigawa R., Iizuka M., Iida Y. (2021). Randomised clinical trial: Pemafibrate, a novel selective peroxisome proliferator-activated receptor α modulator (SPPARMα), versus placebo in patients with non-alcoholic fatty liver disease. Aliment. Pharmacol. Ther..

[30] Kob R., Bollheimer L.C., Bertsch T., Fellner C., Djukic M., Sieber C.C., Fischer B.E. (2015). Sarcopenic obesity: Molecular clues to a better understanding of its pathogenesis?. Biogerontology.

[31] Araki M., Nakagawa Y., Oishi A., Han S.I., Wang Y., Kumagai K., Ohno H., Mizunoe Y., Iwasaki H., Sekiya M. (2018). The Peroxisome Proliferator-Activated Receptor α (PPARα) Agonist Pemafibrate Protects against Diet-Induced Obesity in Mice. Int. J. Mol. Sci..

[32] Smati S., Polizzi A., Fougerat A., Ellero-Simatos S., Blum Y., Lippi Y., Regnier M., Laroyenne A., Huillet M., Arif M. (2022). Integrative study of diet-induced mouse models of NAFLD identifies PPARα as a sexually dimorphic drug target. Gut.

